# Neuroblastoma Cell Lines Are Refractory to Genotoxic Drug-Mediated Induction of Ligands for NK Cell-Activating Receptors

**DOI:** 10.1155/2018/4972410

**Published:** 2018-04-01

**Authors:** Irene Veneziani, Elisa Brandetti, Marzia Ognibene, Annalisa Pezzolo, Vito Pistoia, Loredana Cifaldi

**Affiliations:** ^1^Department of Pediatric Hematology and Oncology, Bambino Gesù Children's Hospital, IRCCS, Rome, Italy; ^2^Laboratorio Cellule Staminali Post Natali e Terapie Cellulari, Istituto Giannina Gaslini, Genova, Italy; ^3^Immunology Research Area, Bambino Gesù Children's Hospital, IRCCS, Rome, Italy

## Abstract

Neuroblastoma (NB), the most common extracranial solid tumor of childhood, causes death in almost 15% of children affected by cancer. Treatment of neuroblastoma is based on the combination of chemotherapy with other therapeutic interventions such as surgery, radiotherapy, use of differentiating agents, and immunotherapy. In particular, adoptive NK cell transfer is a new immune-therapeutic approach whose efficacy may be boosted by several anticancer agents able to induce the expression of ligands for NK cell-activating receptors, thus rendering cancer cells more susceptible to NK cell-mediated lysis. Here, we show that chemotherapeutic drugs commonly used for the treatment of NB such as cisplatin, topotecan, irinotecan, and etoposide are unable to induce the expression of activating ligands in a panel of NB cell lines. Consistently, cisplatin-treated NB cell lines were not more susceptible to NK cells than untreated cells. The refractoriness of NB cell lines to these drugs has been partially associated with the abnormal status of genes for ATM, ATR, Chk1, and Chk2, the major transducers of the DNA damage response (DDR), triggered by several anticancer agents and promoting different antitumor mechanisms including the expression of ligands for NK cell-activating receptors. Moreover, both the impaired production of reactive oxygen species (ROS) in some NB cell lines and the transient p53 stabilization in response to our genotoxic drugs under our experimental conditions could contribute to inefficient induction of activating ligands. These data suggest that further investigations, exploiting molecular strategies aimed to potentiate the NK cell-mediated immunotherapy of NB, are warranted.

## 1. Introduction

Neuroblastoma (NB), the most aggressive extracranial solid tumor occurring in childhood, remains a challenging malignancy [1]. In spite of intensive therapeutic strategies based on surgery, radiotherapy, chemotherapy, and differentiating treatment with 13-*cis*-retinoic acid, NB outcome remains poor, with 3-year event-free survival lower than 40%. In particular, systemic chemotherapy administered to all patient groups (low, intermediate, and high risk), before surgery (neoadjuvant chemotherapy) or after surgery (adjuvant chemotherapy), represents the main treatment [2]. Of note, several genotoxic drugs, including those used in the treatment of NB, cause many side effects [3]. Thus, in order to minimize drug toxicity and to ameliorate the outcome and the lifestyle of the little patients affected by NB, further therapeutic options are needed.

The NK cell-based immunotherapy of cancer, including NB, is attracting more and more interest [4, 5]. The success of this approach depends on the susceptibility of NB cells to the cytolytic activity of allogeneic or autologous ex vivo expanded and activated, adoptively transferred, mature NK cells [6]. The antitumor efficacy of NK cells can be impaired by various immune-escape mechanisms adopted by high-risk NB cells. Thus, NB cells downregulate the expression of MHC class I molecules, rendering tumor cells resistant to T cell lysis [7], and display a reduced expression of ligands for NK cell-activating receptors whereby they become refractory also to the recognition and lysis operated by NK cells [8, 9]. Of note, the susceptibility of cancer cells to NK cell-mediated recognition and lysis is strictly dependent on the expression of ligands for NK cell-activating receptors [10]. Thus, the restoration of the expression levels of these ligands could represent a strategic approach to enhance NB susceptibility to the immune system.

The genotoxic effect typical of several anticancer agents induces DNA damage response (DDR), the pathway most extensively associated with the induction of the expression of ligands for NK cell-activating receptors [11–14]. Ataxia-telangiectasia mutated kinase (ATM) is recruited and activated by DNA double-strand breaks and, through the activation of Chk2 checkpoint kinase [15], is involved in several pathways including the stabilization and activation of p53 [16–18]. p53 is known to induce the expression of ULBP1 and ULBP2 [19, 20], two ligands for the NKG2D NK cell receptor. ATM and Rad3-related (ATR), triggered by DNA single-strand breaks through the activation of Chk1 checkpoint kinase [21], induces cell cycle arrest and senescence, conditions promoting the induction of ligands for NK cell-activating receptors [13, 22, 23]. Moreover, the genotoxic drug-induced ROS generation has been associated with the expression of activating ligands in tumor cells [24–26].

Of note, several chemotherapeutic agents have been reported to function as potent immune adjuvants [27] and to be able to induce the expression of ligands for NK cell-activating receptors in tumor cells of different origin, thus rendering these latter cells more susceptible to NK cell-mediated cytotoxicity [28–30].

To investigate whether DDR caused by genotoxic drugs in NB cells could affect the expression of ligands for NK cell-activating receptors, we tested the effect of chemotherapeutic agents currently used in the treatment of NB. Differently to the reported effect of several drugs on different tumor cell lines [29, 30], our data show that cisplatin, etoposide, irinotecan, and topotecan did not induce the expression of activating ligands in a panel of NB cell lines. Moreover, the status of *ATM*, *ATR*, *CHEK1*, and *CHEK2* genes evaluated in these cell lines was partially altered, suggesting an impaired DDR pathway. Of note, other authors reported that the loss of *ATM* in NB patient samples was associated with poor prognosis [31]. Interestingly, *ATM* maps on chromosome 11q whose hemizygous deletion, together with the loss of chromosome 1p [32], the gain of chromosome 17q [33], and the amplification of both *MYCN* [34] and *ALK* [35, 36], is a well-established marker of NB poor prognosis. Of note, some NB cell lines were unable to produce ROS and to undergo stabilization of p53 levels in response to genotoxic drugs, thus contributing to the impaired induction of activating ligand expression.

The NB refractoriness in response to these genotoxic agents, in terms of induction of activating ligands, suggests that these drugs do not function as immune adjuvants and, thus, could not support the NK cell-mediated recognition and lysis of tumor cells. In order to boost NK cell-based immunotherapy of NB, the effect of different molecules should be more extensively investigated.

## 2. Materials and Methods

### 2.1. Cell Lines and Drugs

Human NB cell lines were obtained as follows: SK-N-AS, SH-SY5Y, SH-EP, SK-N-SH, SK-N-BE(2)c, and IMR-32 from the American Type Culture Collection (ATCC) and LA-N-5 from the Leibniz-Institut DSMZ. All NB cell lines were characterized by (i) HLA class I typing by PCR-SSP sets (Genovision) according to the instructions of the manufacturer and (ii) array comparative genomic hybridization (a-CGH) and single-nucleotide polymorphism (SNP) array analyses (see below). The human non-small-cell lung cancer cell line A549 was purchased from Sigma-Aldrich. The human erythroleukemia cell line K562 was purchased from ATCC and used as a control target for NK cell functional assays. Cells were grown in RPMI 1640 medium supplemented with 10% FBS (Thermo Fisher Scientific), 2 mM glutamine, 100 mg/ml penicillin, and 50 mg/ml streptomycin (EuroClone S.p.A.). Cisplatin (Accord Healthcare Limited), etoposide (Teva Italia), irinotecan (Campo, Pfizer), and topotecan (GlaxoSmithKline) were kindly provided by the pharmacy of our institution.

### 2.2. Antibodies, Flow Cytometry, Western Blotting, and ROS Production

The following antibodies for flow cytometry were used: anti-CD107a-FITC (H4A3), anti-CD3-Alexa-700 (UCHT1), anti-CD56-PE-Cy7 (B159), and anti-CD45 (HI30), purchased from BD Biosciences; anti-ULBP1-PE (170818), anti-ULBP2/5/6-PE (165903), anti-ULBP3-PE (166510), anti-MICA (159227), anti-MICB (236511), anti-TRAIL/R2-APC (17908), anti-CD155/PVR-PE (300907), and anti-Nectin-2/CD112-APC (610603), purchased from R&D Systems; W6/32 which recognizes human fully assembled MHC class I heavy chains; and goat F(ab′)2 Fragment anti-mouse IgG FITC (IM1619, Dako) for flow cytometry. Apoptosis of tumor cells was evaluated with APC-conjugated AnnexinV (BD-Pharmingen) and propidium iodide (PI) (Sigma-Aldrich).

Flow cytometry was performed on FACSCantoII and analysed by FACSDiva Software (BD Biosciences).

ROS production was evaluated in drug-treated NB cell lines by using CellROX Deep Red Reagent (C10422, Invitrogen) and measured by flow cytometry.

Whole-cell extracts were quantified by a bicinchoninic acid assay (Thermo Fisher Scientific), resolved on 8–10% SDS-PAGE and electroblotted. Filters were probed with primary antibodies followed by goat anti-mouse and HRP-conjugated rabbit anti-goat IgG (Jackson). The following antibodies for Western blotting were used: anti-p53 (FL-393) and anti-actin (I-19), purchased by Santa Cruz Biotechnology.

### 2.3. Genomic Profile of NB Cell Lines

DNA from NB cell lines was tested by the high-resolution a-CGH and SNP arrays using the 4 × 180K kit (Agilent Technologies) with a mean resolution of approximately 40 kb. SNP-array and oligoarray data were analysed with Genomic Workbench 7.0.40 software (Agilent). Chromosome positions were determined using GRCh/hg19 (UCSC Genome Browser, http://genome.ucsc.edu, Feb. 2009 release). The quality of the test was assessed on the strength of the QCmetrics values. Polymorphisms (http://dgv.tcag.ca/dgv/app/home) were not included because they were considered normal variants.

### 2.4. NK Cell Isolation

Human NK cells were isolated from peripheral blood mononuclear cells (PBMCs) of healthy donors with the RosetteSep NK cell-enrichment mixture method (StemCell Technologies) and Ficoll-Paque Plus (Lympholyte Cedarlane) centrifugation. NK cells were routinely checked for the CD3^−^CD56^+^ immunophenotype by flow cytometry, and those with purity greater than 90% were cultured with 200 IU/ml of recombinant human IL-2 (PeproTech) at 37°C and tested up to 5 days after isolation.

### 2.5. NK Cell Degranulation Assay

A degranulation assay was performed by coculturing NK cells with target cells at a 1 : 1 ratio for K562 and a 1 : 2 ratio for A562 and NB cell lines, for 3 hours, in complete medium, in the presence of anti-CD107a, and in the last 2 hours of GolgiStop (BD Biosciences). Then, cells were stained with anti-CD3, anti-CD56, and anti-CD45, and the expression of CD107a was evaluated by flow cytometry in the CD3^−^CD56^+^CD45^+^ subset.

### 2.6. Statistical Analysis

Data values were evaluated by two-tailed paired Student's *t*-test. Normalized values were analysed for correlation by the regression analysis using GraphPad software. *p* values not exceeding 0.05 were considered to be statistically significant.

## 3. Results

### 3.1. Drugs Used for NB Treatment Did Not Induce the Expression of Ligands for NKG2D- and DNAM1-Activating Receptors on NB Cell Lines

**Figure 1:**
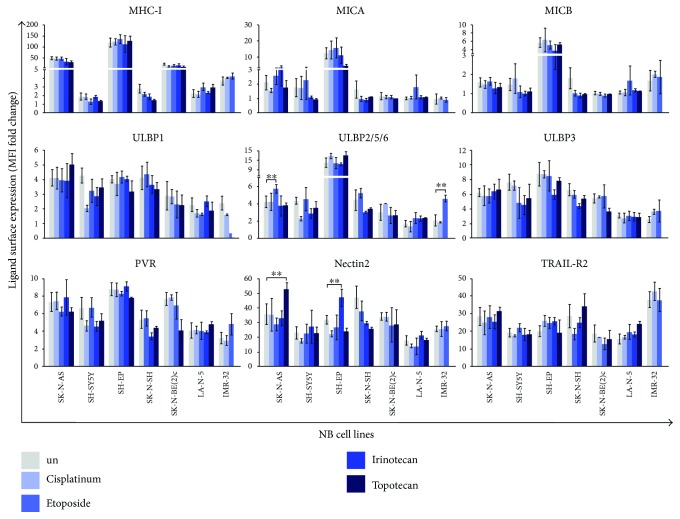
Anticancer drugs do not affect the expression of ligands for NK cell-activating receptors in NB cell lines. The NB cell lines SK-N-AS, SH-SY5Y, SH-EP, SK-N-SH, SK-N-BE(2)c, LA-N-5, and IMR-32 were untreated (un) or treated with cisplatin (at 2 *μ*M), etoposide (at 0.1 *μ*M), irinotecan (at 1 nM), and topotecan (at 10 nM) for 24 hours, and the surface expression of each indicated ligands for NK cell-activating receptors was analysed by flow cytometry. The surface expression of activating ligands was expressed as mean fluorescence intensity (MFI) that was normalized to MFI of IgG isotype Ab used as control (MFI fold change). The mean ± SD of MFI fold changes obtained by 5 independent stainings of each drug-treated NB cell line are reported in histograms (two-tailed paired Student's *t*-test) (^∗∗^ *p* < 0.01).

We investigated whether drugs used in the treatment of NB could affect the expression of ligands for NK cell-activating receptors. The genotoxic drugs cisplatin (DNA binder), etoposide (topoisomerase II inhibitor), irinotecan, and topotecan (topoisomerase I inhibitors) were used to treat *in vitro* the following NB cell lines: SK-N-AS, SH-SY5Y, SH-EP, SK-N-SH, SK-N-BE(2)c, LA-N-5, and IMR-32. Of note, the status of p53, a key regulator of the induction of some NKG2D ligands [19, 20], is wild type in all NB cell lines used with the exception of SK-N-AS and SK-N-BE(2)c in which p53 is lost due to a gene mutation, as we previously reported [9]. These cell lines were cultured for 24 and 48 hours in the presence of each drug at different concentrations in order to identify the optimal conditions enabling cell viability and preventing apoptosis that could compromise the detection of ligand surface expression [37]. Cell viability was assessed by cell count (data not shown) and apoptosis by AnnexinV and PI staining (data not shown and Supplementary Figure 1). Cisplatin at 2 *μ*M, etoposide at 0.1 *μ*M, irinotecan at 1 nM, and topotecan at 10 nM induced apoptosis of all NB cell lines after 48 hours of treatment, with the exception of SK-N-AS treated with the first three drugs and SK-N-BE(2)c treated with all four drugs (Supplementary Figure 1). Apoptosis was not detected at an earlier time, indicating that 24-hour drug treatment was the best time point to evaluate the levels of activating ligands. Under these conditions, cisplatin, etoposide, irinotecan, and topotecan did not induce the expression of MHC class I molecule ligands for activating killer-immunoglobulin-like receptors (KIRs), MICA, MICB, ULBP1, ULBP2/5/6, ULBP3 ligands for NKG2D, PVR, and Nectin2 ligands for DNAM1 and TRAIL-R2 interacting with TRAIL (Figure 1). Some exceptions were found for the induction of ULBP2/5/6 on SK-N-AS and IMR-32 by etoposide and of Nectin2 on SK-N-AS and SH-EP by topotecan and irinotecan, respectively.

These data suggest that four drugs commonly used for chemotherapy of NB patients, that is, cisplatin, etoposide, irinotecan, and topotecan, induce apoptosis of p53 wild-type NB cell lines and, at preapoptotic concentrations, do not induce the expression of activating ligands. Thus, these drugs do not exert immune adjuvant effects on antitumor NK cell-mediated functions.

### 3.2. ATM, ATR, CHEK1, and CHEK2 Status on NB Cell Lines

**Figure 2:**
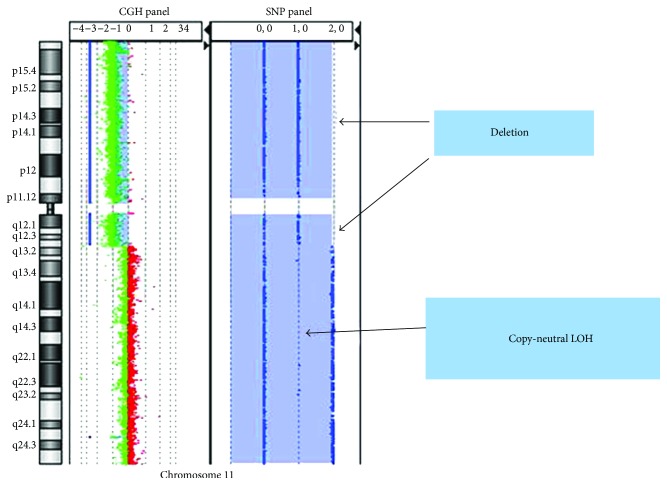
Status of chromosome 11 in the SK-N-BE(2)c cell line: copy-neutral LOH region in chromosome 11q13.2–q24.3 containing *ATM* and *CHEK1* genes. Right panel Log2 ratio data and aberration calls on chromosome 11q (green dots) based on data from the CGH probes on the a-CGH + SNP array. Left panel SNP copynumber calls (blue dots) for SNP sites. An LOH call appears as a large mauve shaded area; note the lack of probes that correspond to a copynumber of heterozygous signals (1, 0) affecting chromosome 11q13.2–q24.3. Two hemizygous deletions (2, 0) affect chromosome 11p15.5–p11.12 and 11q11– q13.1.

To assess if the impaired modulation of activating ligands by the four drugs tested could be related to altered signalling upon drug-mediated genotoxic DNA damage, the status of genes encoding ATM (*ATM*), ATR (*ATR*), Chk1 (*CHEK1*), and Chk2 (*CHEK2*), the main molecules involved in DDR [38], was analysed in our panel of NB cell lines (Table 1). a-CGH revealed that the *ATM* gene was normal in SH-SY5Y, SK-N-SH, and LA-N-5 but was lost in SK-N-AS, SH-EP, and IMR-32. By contrast, the status of the *ATR* gene was normal in all NB cell lines with the exception of SK-N-AS and SH-EP in which *ATR* gain was detected. In addition, the status of genes for checkpoint kinases was shown to be partially altered: *CHEK1* was lost in both SK-N-AS and IMR-32, *CHEK2* was lost in both SK-N-AS and SK-N-BE(2)c, and both were normal in all other cell lines. Moreover, *ATM* and *CHEK1* were subjected to copy-neutral loss of heterozygosity (cnLOH) in SK-N-BE(2)c (Table 1). cnLOH represents one example of genomic abnormality in which no change in chromosomal copy number occurs and is often associated with resistance to standard therapeutic modalities and poor survival in tumors [39]. Interestingly, SK-N-BE(2)c showed a large region of cnLOH (79.7 Mb) that included *ATM* and *CHEK1* genes (Figure 2). Thus, cnLOH can lead to an effective knockout of gene expression.

These data suggest that the impaired induction of the expression of activating ligands upon DDR was due at least in part to the loss of *ATM*, *CHEK1*, and *CHEK2* and the gain of *ATR* in NB cell lines tested. Additional molecules involved in the DDR pathway triggered by genotoxic drugs and leading to defective modulation of activating ligand expression in NB cells should be investigated.

### 3.3. ROS Production and p53 Stabilization in NB Cell Lines upon Cisplatin, Etoposide, Irinotecan, and Topotecan Treatment

**Figure 3:**
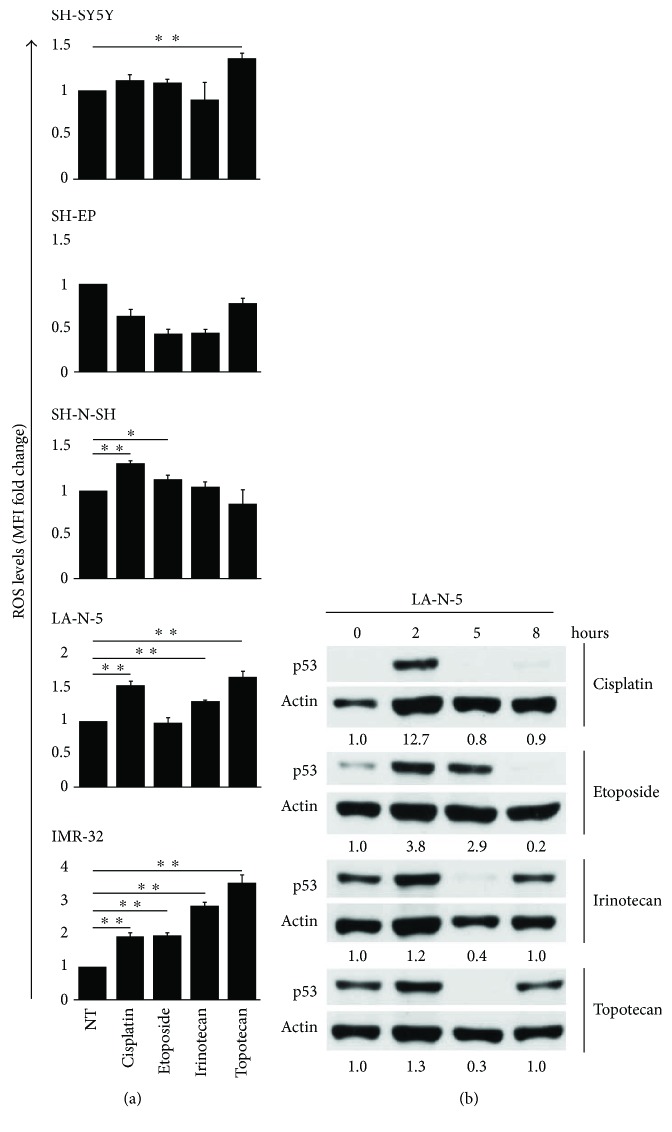
ROS production and p53 stabilization in drug-treated NB cell lines. (a) NB cell lines were untreated (NT) or treated for 24 hours with cisplatin, etoposide, irinotecan, and topotecan, at the same concentrations described in Figure 1, and ROS production was measured by flow cytometry. Drug-induced ROS levels were expressed as MFI normalized to MFI of untreated cells used as control (MFI fold change). The mean ± SD of MFI fold changes obtained by 4 independent stainings of each drug-treated NB cell line are reported in histograms (two-tailed paired Student's *t*-test) (^∗^ *p* < 0.05, ^∗∗^ *p* < 0.01). (b) LA-N-5 was untreated (0) or treated with the same drugs at the same doses described above for 2, 5, and 8 hours, and levels of p53 were measured by immunoblot analysis. An anti-actin Ab was used for normalization. Densitometric analysis of p53 normalized to actin and relative to untreated cells (point 0) are reported below each immunoblot. A representative out of two independent experiments performed is shown.

The production of ROS in genotoxic drug-treated tumor cells has been associated with the induction of NKG2D and DNAM1 ligands [24–26]. Then, p53 wild-type NB cell lines SH-SY5Y, SH-EP, SK-N-SH, LA-N-5, and IMR-32 were treated for 24 hours with cisplatin, etoposide, irinotecan, and topotecan at the above-mentioned concentrations, and ROS levels were measured by flow cytometry (Figure 3(a)). Both LA-N-5 and IMR-32 produced ROS upon incubation with the four drugs, with the exception of LA-N-5 after etoposide treatment. SK-N-SH produced ROS after cisplatin and etoposide but not irinotecan and topotecan treatment. SH-SY5Y produced ROS only after topotecan treatment, whilst SH-EP was completely refractory to generate ROS upon the treatment of all drugs used.

Wild-type p53 expression in tumor cells is generally either undetectable or very low but increases upon exposure to DNA-damaging agents, as a result of its stabilization and activation [17, 18]. Of note, p53 activation occurs rapidly in response to DDR induced by genotoxic drugs and is carried out by ATM. Moreover, p53 has been reported as a transcription factor for some ligands of the NKG2D receptor [19, 20]. Then, to assess if our genotoxic drugs at the doses reported above induced the stabilization of p53 in NB cell lines, the p53 wild type, *ATM* single copy LA-N-5 NB cell line was treated with cisplatin, etoposide, irinotecan, or topotecan for 2, 5, and 8 hours, and the level of p53 was evaluated by Western blotting. LA-N-5 cells showed higher levels of p53 2 hours after drug treatment, particularly evident after cisplatin and etoposide treatment that returned to normal levels at latter time points (Figure 3(b)).

These data indicate that the impaired induction of ligands for NK cell-activating receptors in NB cell lines upon preapoptotic doses of genotoxic drugs could depend also on the abnormal production of ROS and on the transient rather than persistent p53 stabilization.

### 3.4. Cisplatin Does Not Render NB Cell Lines More Susceptible to NK Cell-Mediated Recognition

**Figure 4:**
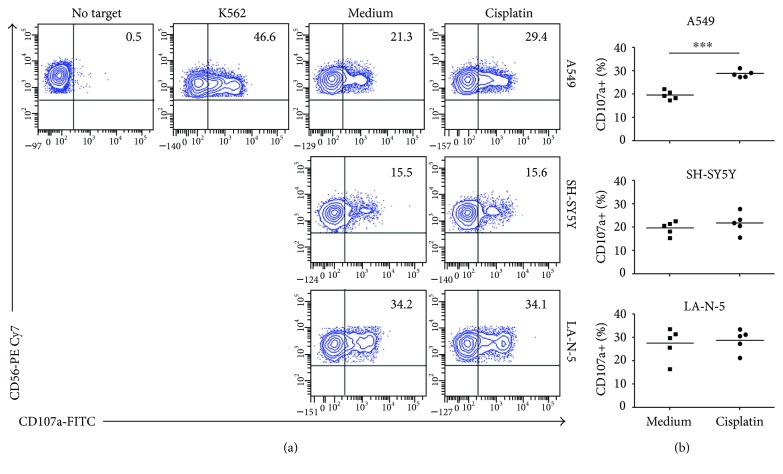
Cisplatin does not sensitize NB cell lines to NK cell recognition. The NB cell lines SH-SY5Y and LA-N-5 were untreated (medium) or treated with 2 *μ*M of cisplatin for 24 hours and used as targets in an NK cell degranulation assay, measured as the CD107a cell-surface expression on human CD3^−^CD56^+^CD45^+^ NK cells from healthy donors. The K562 cell line was used as positive control for NK cell activation. The A549 cell line was untreated or treated with 10 *μ*M of cisplatin for 48 hours and used as positive control for drug effect. (a) A representative experiment out of five performed is shown. The percentage of CD107a^+^ NK cells is indicated. (b) Summaries of NK cell degranulation of NK cells, isolated by five different healthy donors, following stimulation with untreated and cisplatin-treated cell lines are shown. Dots, percentage of CD107a^+^ NK cells; horizontal bars, average values. *p* value compared untreated with cisplatin-treated cell lines (two-tailed paired Student's *t*-test) (^∗∗∗^ *p* < 0.001).

To test whether the impaired, genotoxic drug-dependent modulation of ligands for NK cell-activating receptors in NB cell lines affected NK cell-mediated recognition, we performed an NK cell degranulation assay by using drug-treated NB cell lines as target cells. Cisplatin was previously reported to induce the MICA and MICB expression in non-small-cell lung cancer A549 cell line, through DNA stress-induced ATM-ATR signalling, resulting in enhanced sensitivity to NK cell-mediated cytotoxicity [40]. Therefore, we tested the A549 cell line as a positive control to compare the susceptibility of cisplatin-treated SH-SY5Y and LA-N-5 NB cell lines to NK cell-mediated recognition. We treated A549 with cisplatin at 10 *μ*M for 24 and 48 hours as previously reported [40]. First, we confirmed that 10 *μ*M cisplatin did not induce apoptosis of the A549 cell line after 24 or 48 hours of culture (data not shown). Then, we analysed the expression levels of ligands for NK cell-activating receptors in the cisplatin-treated A549 cell line. At 48 hours of treatment, the MICA and MICB expression was enhanced, as previously reported [40], as well as that of ULBP1, ULBP2/5/6, ULBP3, and PVR (data not shown). Consequently, we performed an NK cell degranulation assay by using as target the A549 cell line treated with 10 *μ*M of cisplatin for 48 hours and both SH-SY5Y and LA-N-5 cell lines treated with 2 *μ*M of cisplatin for 24 hours. The cisplatin-treated A549 cell line was significantly more susceptible to NK cell recognition than untreated cells, as previously reported [40]. In contrast, cisplatin-treated SH-SY5Y and LA-N-5 NB cell lines induced NK cell degranulation comparably to untreated cells (Figure 4).

These data confirm that genotoxic drugs such as cisplatin do not render NB cell lines more susceptible to NK cell recognition.

## 4. Conclusions

Chemotherapy is the antitumor intervention most commonly employed for several cancers including NB [1, 2]. The antitumor action is associated with genotoxic effects leading to DDR caused by many chemotherapeutic agents. The DDR signaling pathway induces apoptosis and cell cycle arrest dependent on the induction of ATM and ATR, respectively [16, 21, 38]. The ATM/ATR pathways are under control of two main checkpoint kinases, that is, Chk1 and Chk2 [15, 21], and, through the activation of the master transcription factor p53, lead to cell senescence and induce the expression of ligands for NK cell-activating receptors via mechanisms that are still under investigation [13, 19, 20, 22, 23, 41]. The induced expression of such ligands renders cancer cells more vulnerable to NK cell lysis [10]. Thus, anticancer agents able to induce the expression of activating ligands are promising candidates to boost NK cell-mediated immunotherapy [30].

Multiagent chemotherapy represents the conventional therapy for NB patients, especially for those at an advanced disease stage. Depending on the risk group, NB chemotherapy can be associated with surgical tumor removal, myeloablative therapy followed by hematopoietic stem cell infusion, and the use of differentiating agents such as 13-*cis*-retinoic acid [1].

Presently, the *in vivo* adoptive transfer of mature *in vitro* activated and expanded NK cells, in autologous or allogeneic settings, represents a promising approach to treat NB. Different clinical trials based on the use of mature NK cells in combination with some chemotherapeutic agents, such as topotecan, are in the recruiting phase as reported on the http://ClinicalTrials.gov website. In order to evaluate whether drugs such as cisplatin, etoposide, irinotecan, and topotecan could sustain the NK cell-mediated antitumor function, we studied the expression of ligands for NK cell-activating receptors on a panel of NB cell lines. No drugs showed a significant immune adjuvant effect confirmed also by an NK cell functional assay upon cisplatin treatment. This refractoriness was partially related to the abnormal status of *ATM*, *ATR*, *CHEK1*, and *CHEK2* genes in the NB cell lines tested. Of note, *ATM* deletion correlates with lower event-free survival and overall survival in patients affected by NB, independently of *MYCN* amplification [31]. Thus, NB cells bearing *ATM* deletion could often result in resistance to chemotherapy due to an impaired DDR pathway and, consequently, to an inefficient apoptosis or cell cycle arrest. The altered ATM/ATR induction in NB cells could inhibit the effect mediated by many genotoxic drugs in terms of the induced expression of ligands for NK cell-activating receptors. Moreover, our data showed that both the impaired production of ROS in some NB cell lines and the transient p53 stabilization after drug treatment at preapoptotic concentrations could contribute to inefficient induction of NKG2D and DNAM1 ligands. It cannot be excluded that in our drug treatment conditions the apoptosis induced at 48 hours and at higher doses could depend also on p53-independent mechanisms occurring in NB, as reported by other authors [42, 43]. A note of caution comes from the fact that all the experiments were performed using a panel of NB cell lines, and no information is available on primary NB cells.

In conclusion, based on these data and concepts, in order to boost the NK cell-mediated immunotherapy of NB, the combined use of immune adjuvant agents could represent a winning approach. Thus, the effect of molecules efficiently inducing the expression of ligands for NK cell-activating receptors on NB cells remains to be evaluated.

## Figures and Tables

**Table 1:** Status of *ATM*, *ATR*, *CHEK1*, and *CHEK2* genes in NB cells.

NB cell lines	*ATM* gene	Chromosomal coordinates of *ATM* loss/cnLOH	*ATR* gene	Chromosomal coordinates of *ATR* gain	*CHEK1* gene	Chromosomal coordinates of *CHEK1* loss/cnLOH	*CHEK2* gene	Chromosomal coordinates of *CHEK2* loss
SK-N-AS	Loss	Chr11: 72159638–134720344 Cytoband: 11q13.4–q25 Size: 62.5 Mb	Gain	Chr3: 94300781–197840323 Cytoband: 3q11.2–q29 Size: 103.5 Mb	Loss	Chr11: 72159638–134720344 Cytoband: 11q13.4–q25 Size: 62.5 Mb	Loss	Chr22: 23283056–31436822 Cytoband: 22q11.22–q12.2 Size: 8.1 Mb
SH-SY5Y	Single copy	—	Single copy	—	Single copy	—	Single copy	—
SH-EP	Loss	Chr11: 93455106–119038765 Cytoband: 11q21–q23.3 Size: 25.6 Mb	Gain	Chr3: 133671502–197801441 Cytoband: 3q22.1–q29 Size: 64.1 Mb	Single copy	—	Single copy	—
SK-N-SH	Single copy	—	Single copy	—	Single copy	—	Single copy	—
SK-N-BE(2)c	Copy-neutral LOH	Chr11: 55196818–134924542 Cytoband: 11q13.2–q24.3 Size: 79.7 Mb	Single copy	—	Copy neutral LOH	Chr11: 55196818–134924542 Cytoband: 11q13.2–q24.3 Size: 79.7 Mb	Loss	Chr22: 16153099–51224252 Cytoband: 22q11.1–q13.33 Size: 35.0 Mb
LA-N-5	Single copy	—	Single copy	—	Single copy	—	Single copy	—
IMR-32	Loss	Chr11: 85989063–134446160 Cytoband: 11q14.2–q25 Size: 48.4 Mb	Single copy	—	Loss	Chr11: 85989063–134446160 Cytoband: 11q14.2–q25 Size: 48.4 Mb	Single copy	—

## Supplementary Materials

Supplementary Materials Supplementary Figure 1: apoptotic state of NB cell lines upon genotoxic drug treatment. NB cell lines were untreated (NT) or treated (drug) with 2 *μ*M of cisplatin, 0.1 *μ*M of etoposide, 1 nM of irinotecan, and 10 nM of topotecan for 24 and 48 hours. Apoptosis was measured by AnnexinV and PI staining and flow cytometry analysis. The percentage of cells in the early phase of apoptosis (AnnexinV^+^PI^−^, blue bar) and in the late phase of apoptosis (AnnexinV^+^PI^+^, orange bar) and those of dead cells (AnnexinV^−^PI^+^, gray bar) are indicated in the stacked histogram. A representative experiment out of three performed is shown.
