# Supplementary figures and images for: Neuroblastoma Cell Lines Are Refractory to Genotoxic Drug-Mediated Induction of Ligands for NK Cell-Activating Receptors
Source: J Immunol Res. 2018 Apr 1;2018:4972410. doi: 10.1155/2018/4972410 (PMC5901817; doi:10.1155/2018/4972410)

Supplementary Figure 1

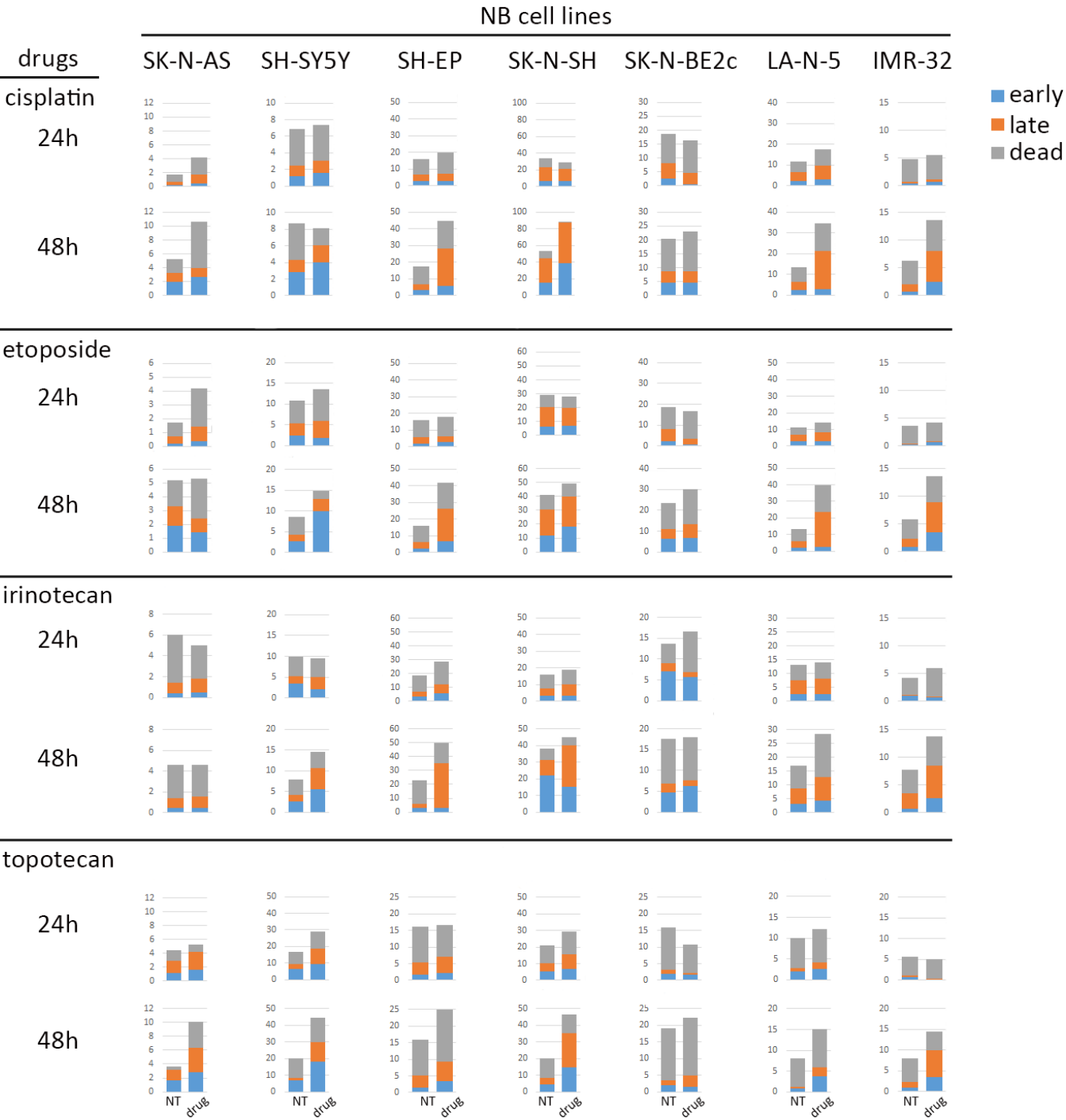

Supplement: Supplementary Materials — Supplementary Figure 1: apoptotic state of NB cell lines upon genotoxic drug treatment. NB cell lines were untreated (NT) or treated (drug) with 2 μM of cisplatin, 0.1 μM of etoposide, 1 nM of irinotecan, and 10 nM of topotecan for 24 and 48 hours. Apoptosis was measured by AnnexinV and PI staining and flow cytometry analysis. The percentage of cells in the early phase of apoptosis (AnnexinV+PI−, blue bar) and in the late phase of apoptosis (AnnexinV+PI+, orange bar) and those of dead cells (AnnexinV−PI+, gray bar) are indicated in the stacked histogram. A representative experiment out of three performed is shown. [file 4972410.f1.pdf]
